# Global Perspectives on HPV Vaccination: Achievements, Challenges, and Lessons from the Brazilian Experience

**DOI:** 10.3390/vaccines13111106

**Published:** 2025-10-29

**Authors:** Antonio Braga, Caroline Alves de Oliveira Martins, Gabriela Paiva, Érica de Almeida Barboza, Marcela Chagas, Gustavo Yano Callado, Edward Araujo Júnior, Jorge de Rezende-Filho, Isabel Cristina Chulvis do Val Guimarães, Roberta Granese, Gloria Calagna, Susana Cristina Aidé Viviani Fialho

**Affiliations:** 1 Department of Gynecology and Obstetrics, School of Medicine, Federal University of Rio de Janeiro, Rio de Janeiro 22240-003, RJ, Brazil; bragamed@yahoo.com.br (A.B.); gabrielapaivaslc@gmail.com (G.P.); rezendef@me.ufrj.br (J.d.R.-F.); 2 Department of Maternal and Child Health, School of Medicine, Fluminense Federal University, Niterói 24070-090, RJ, Brazil; carolineaom@id.uff.br (C.A.d.O.M.); isabelval@id.uff.br (I.C.C.d.V.G.); susanaaide@id.uff.br (S.C.A.V.F.); 3 Postgraduate Program in Applied Health Sciences, University of Vassouras, Vassouras 27700-000, RJ, Brazil; erica_burk@hotmail.com (É.d.A.B.); marcelavchagas@gmail.com (M.C.); 4 Faculdade Israelita de Ciências da Saúde Albert Einstein, Hospital Israelita Albert Einstein, São Paulo 05652-900, SP, Brazil; gycallado@gmail.com; 5 Department of Obstetrics, Paulista School of Medicine, Federal University of São Paulo (EPM-UNIFESP), São Paulo 04023-062, SP, Brazil; araujojred@terra.com.br; 6 Discipline of Woman Health, Municipal University of São Caetano do Sul (USCS), São Caetano do Sul 09521-160, SP, Brazil; 7 Department of Biomedical and Dental Sciences and Morphofunctional Imaging, “G. Martino” University Hospital, 98100 Messina, Italy; rgranese@unime.it; 8 Obstetrics and Gynecology Unit, Villa Sofia Cervello Hospital, University of Palermo, 90100 Palermo, Italy

**Keywords:** HPV vaccine, cervical cancer, vaccine uptake, vaccine hesitancy, Brazil, global health

## Abstract

Background: The introduction of prophylactic HPV vaccination has transformed cervical cancer prevention worldwide, yet many low- and middle-income countries face persistent challenges in implementation, coverage gaps, and vaccine hesitancy. This article presents a narrative review of global and Brazilian HPV vaccination programs, highlighting achievements, pitfalls, and lessons for future strategies. Methods: We reviewed peer-reviewed literature and official reports from WHO, PAHO, CDC, Brazilian institutions, and others, focusing on programmatic performance, coverage trends, and vaccine acceptance. Results: In high-income settings such as Australia and the United Kingdom, school-based vaccination programs have driven sharp declines in HPV prevalence, genital warts, and precancerous lesions, in some cases approaching elimination thresholds. The United States has made progress but continues to struggle with disparities in uptake linked to socioeconomic and cultural factors. In India and several African nations, recent evidence supports single-dose regimens as a cost-effective and logistically feasible strategy. In Brazil, HPV vaccination was introduced in 2014 via the National Immunization Program (PNI), initially targeting girls aged 9–13 years through school campaigns. First-dose coverage exceeded 80% in the first year but subsequently declined, with full-schedule completion rates dropping below 60%. Contributing factors include misinformation, weakening of school-based delivery, and pandemic-related disruptions. Brazil later expanded eligibility to boys and immunocompromised populations and, more recently, extended catch-up vaccination to older adolescents. Conclusions: HPV vaccination has the potential to substantially reduce cervical cancer incidence globally. However, sustained impact depends not only on infrastructure and universal access but also on consistent school-based delivery, adaptive policies such as single-dose regimens, and robust communication strategies to counter misinformation. Brazil’s experience offers both inspiration and caution, providing lessons for countries striving to meet the WHO 90-70-90 targets.

## 1. Introduction

Cervical cancer remains a major cause of morbidity and mortality worldwide, with updated GLOBOCAN 2022 estimates reporting the burden across 185 countries [1]. These data highlight persistent inequities, with the highest incidence and mortality in low- and middle-income regions. It remains a major global health challenge, with an estimated 600,000 new cases and over 340,000 deaths annually [2]. Persistent infection with high-risk human papillomavirus (HPV), particularly types 16 and 18, is the primary causal factor in nearly all cases of cervical cancer [3].

Cervical cancer is an invasive malignant neoplasm that arises from untreated or persistent high-grade cervical intraepithelial neoplasia (CIN), especially CIN grade 3, which represents a premalignant alteration of the cervical epithelium [4]. It is the fourth most common cancer among individuals with a cervix worldwide, and its burden is disproportionately concentrated in low- and middle-income countries (LMICs), where limited access to effective screening and vaccination programs exacerbates outcomes [5].

The development of HPV vaccines is the culmination of decades of basic and translational research linking papillomavirus infection to cervical cancer. In the late 1970s and 1980s, the pioneering work of Harald zur Hausen established that persistent infection with oncogenic HPV types is the necessary cause of cervical cancer, challenging prevailing theories that herpes simplex virus might play this role. His discovery that HPV16 and HPV18 DNA were consistently present in cervical cancer cells laid the foundation for targeted prevention. Building on these insights, Ian Frazer and colleagues in Australia developed virus-like particle (VLP) technology in the 1990s, which enabled the creation of safe, highly immunogenic vaccines that mimic the viral capsid without containing viral DNA. These milestones culminated in the first licensing of prophylactic HPV vaccines in 2006, revolutionizing cancer prevention. In recognition of this paradigm-shifting discovery, Harald zur Hausen was awarded the 2008 Nobel Prize in Physiology or Medicine, underscoring the global significance of HPV research for reproductive health and cancer control [6,7].

Early adopters such as Australia, the United Kingdom, and the United States implemented national vaccination programs that rapidly reduced HPV infection rates, genital warts, and high-grade cervical lesions [8,9]. In LMICs, implementation has lagged; however, growing evidence indicates that with appropriate strategies, meaningful impact is achievable [10].

In Brazil, cervical cancer is the third most common malignancy among individuals with a cervix, and the country ranks fourth worldwide in incidence [11]. According to estimates from the Brazilian National Cancer Institute (Instituto Nacional de Câncer José Alencar Gomes da Silva, INCA) for the 2023–2025 triennium, approximately 17,010 new cases are diagnosed annually, second only to breast cancer (73,610 cases) and colorectal cancer (23,660 cases) [10]. Despite the availability of effective screening programs when well organized, the disease continues to affect many individuals with a cervix worldwide—particularly in developing countries such as Brazil, where structural inequalities, limited coverage, and late presentation contribute to persistently high mortality [12].

In 2020, the World Health Organization launched a global strategy to eliminate cervical cancer as a public health problem—the “90-70-90” targets: 90% of individuals with a cervix vaccinated by age 15, 70% of women screened at least twice, and 90% of those with cervical disease treated appropriately [13]. Brazil has formally adhered to this strategy and, amid both advances and challenges, continues to address the burden of cervical cancer through vaccination, screening, and treatment initiatives.

The COVID-19 pandemic caused significant setbacks for immunization systems worldwide, disrupting school-based HPV delivery and reducing uptake across income settings. In the post-pandemic period, many programs intensified recovery through catch-up campaigns, digital tracking, and integration with broader adolescent-health initiatives. In Brazil, the Ministry of Health adopted a single-dose schedule for ages 9–14 and implemented a national catch-up strategy for adolescents up to 19 years old who missed vaccination during the pandemic, extending eligibility until December 2025 [14]. These measures aim to rebuild herd protection, address coverage gaps, and strengthen program resilience within the National Immunization Program (PNI).

This review was designed to provide a contextual and interpretative synthesis of global and Brazilian experiences with HPV vaccination, integrating data from scientific publications and official public health sources. By combining international evidence with the Brazilian context, it offers an updated overview of achievements, challenges, and lessons learned, aiming to support health policymakers and program managers in improving vaccine coverage, strengthening prevention, and contributing to the global goal of cervical cancer elimination. Rather than performing a quantitative meta-analysis or bibliometric assessment, the review focuses on describing trends, implementation strategies, and policy lessons relevant to cervical cancer prevention.

## 2. Methods

This is a narrative review providing a descriptive and interpretative synthesis of the literature, without protocol registration, risk-of-bias assessment, or meta-analysis. Selected PRISMA 2020 reporting elements were used in an adapted form to enhance transparency in the identification and selection of records, without implying a systematic review protocol [15].

A comprehensive literature search was conducted in three databases—PubMed, Scopus, and Web of Science—for records published between January 2006 and May 2025 in English, Portuguese, or Spanish. Search strategies were adapted to the specific syntax of each database, as follows: PubMed (Title/Abstract and MeSH): (“Papillomavirus Vaccines” [MeSH] OR “HPV vaccine*”) AND (coverage OR uptake OR hesitancy OR implementation) AND (Brazil OR Australia OR “United Kingdom” OR “United States” OR India OR Mexico); Scopus (TITLE-ABS-KEY): (“HPV vaccine*” OR “papillomavirus vaccine*”) AND (coverage OR uptake OR hesitancy OR implementation) AND (Brazil OR Australia OR “United Kingdom” OR “United States” OR India OR Mexico) AND PUBYEAR > 2006, and Web of Science (Topic = TS): (“HPV vaccine*” OR “papillomavirus vaccine*”) AND (coverage OR uptake OR hesitancy OR implementation) AND (Brazil OR Australia OR “United Kingdom” OR “United States” OR India OR Mexico).

A comprehensive literature search was conducted in three databases—PubMed, Scopus, and Web of Science—for records published between January 2006 and May 2025 in English, Portuguese, or Spanish. Search strategies were adapted to the specific syntax of each database, as follows: PubMed (Title/Abstract and MeSH): (“Papillomavirus Vaccines” [MeSH] OR “HPV vaccine*”) AND (coverage OR uptake OR hesitancy OR implementation) AND (Brazil OR Australia OR “United Kingdom” OR “United States” OR Canada OR Spain OR China OR India); Scopus (TITLE-ABS-KEY): (“HPV vaccine*” OR “papillomavirus vaccine*”) AND (coverage OR uptake OR hesitancy OR implementation) AND (Brazil OR Australia OR “United Kingdom” OR “United States” OR Canada OR Spain OR China OR India) AND PUBYEAR > 2006; Web of Science (Topic = TS): (“HPV vaccine*” OR “papillomavirus vaccine*”) AND (coverage OR uptake OR hesitancy OR implementation) AND (Brazil OR Australia OR “United Kingdom” OR “United States” OR Canada OR Spain OR China OR India).

In addition, official reports and policy documents from the World Health Organization (WHO), Pan American Health Organization (PAHO), Centers for Disease Control and Prevention (CDC), the Brazilian Ministry of Health, and others were manually retrieved to capture relevant literature not indexed in bibliographic databases. These institutional sources were used only to supplement contextual and programmatic information.

Eligible materials included peer-reviewed articles, surveillance reports, and programmatic evaluations addressing HPV vaccine introduction, coverage, or barriers in Brazil. Editorials, commentaries, theses, and non-empirical papers were excluded.

Comparator countries were selected according to the availability of national immunization data and representativeness across income levels and world regions—high-income: Australia, United Kingdom, United States, Canada, and Spain; and upper-middle-income: China, Mexico, India, and Brazil, to capture regional diversity and programmatic evolution.

Two reviewers independently screened titles and abstracts, assessed full texts for eligibility, and extracted data on study design, population, vaccine type and schedule, coverage levels, determinants of uptake or hesitancy, and implementation strategies. Because of heterogeneity across designs and outcomes, no meta-analysis was performed; results were summarized descriptively to identify trends and policy gaps.

**Figure 1:**
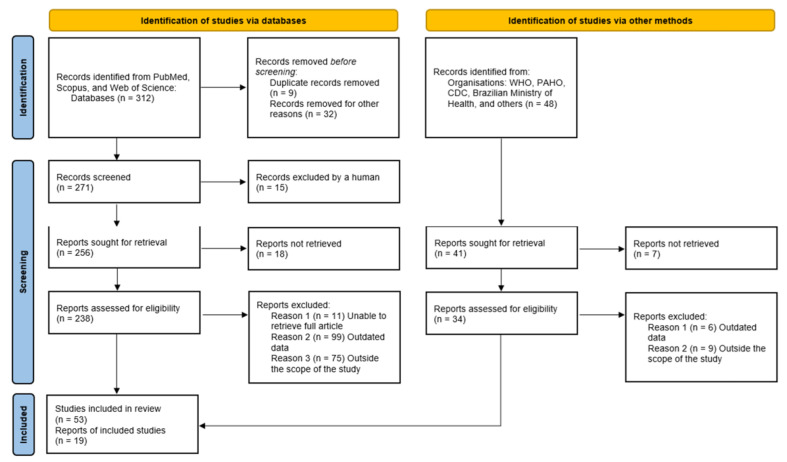
PRISMA 2020 flow diagram (adapted for a narrative review) for study selection [15]. Flow of records through identification, screening, eligibility, and inclusion. After screening and eligibility assessment, *n* = 72 records (peer-reviewed articles and official reports from WHO, PAHO, CDC, the Brazilian Ministry of Health, and others) were included in the qualitative synthesis.

The study selection process is illustrated in Figure 1, following the PRISMA 2020 flow diagram [15].

## 3. Results

### 3.1. Global Progress in HPV Vaccination

Since the licensure of the first HPV vaccines in 2006, a growing number of countries have integrated them into national immunization programs, achieving substantial population-level impact (Table 1). Several prophylactic HPV vaccines are currently licensed worldwide. In addition to Cervarix^®^ (bivalent, HPV16/18; GSK (London, UK)), Gardasil^®^ (quadrivalent, 6/11/16/18; Merck/MSD (Rahway, NJ, USA)), and Gardasil 9^®^ (nonavalent, 6/11/16/18/31/33/45/52/58; Merck/MSD (Rahway, NJ, USA))—all WHO-prequalified—newer vaccines have expanded global options. Cecolin^®^ (bivalent, HPV16/18; Xiamen Innovax, Xiamen, China) received WHO prequalification in 2021 and updated WHOPAR in 2024, supporting its use in single-dose schedules. Walrinvax^®^ (HPV-2; Zerun/Walvax, Shanghai, China) was licensed nationally in 2022 and WHO-prequalified in 2024. Cervavac^®^ (quadrivalent, 6/11/16/18; Serum Institute of India (Pune, India)) has been rolled out in India since 2023 and is currently under WHO PQ evaluation. In 2025, Cecolin-9^®^ (nonavalent; Innovax, Xiamen, China) obtained national marketing authorization, broadening access to multivalent protection. WHO’s 2024 product-choice guidance emphasizes that expanding vaccine supply—including these newer formulations—will be critical to achieving equitable global coverage.

Australia is widely recognized as a pioneer: by introducing school-based vaccination in 2007 with coverage exceeding 80% among adolescent girls, it recorded a dramatic reduction in HPV infection, genital warts, and high-grade cervical intraepithelial neoplasia within a decade [16]. Modeling studies now project that Australia may become the first country to eliminate cervical cancer as a public health problem by 2035, provided that high vaccination and screening coverage are maintained [17].

The United Kingdom offers another model of success. Following the launch of its school-based program in 2008, coverage consistently surpassed 85%, and national surveillance showed an 86% reduction in HPV 16/18 prevalence among individuals under 21 with a cervix [18]. By 2021, robust registry-based analyses documented an almost 90% decline in cervical cancer incidence among those vaccinated at ages 12–13, providing the strongest real-world evidence to date of the vaccine’s cancer-preventive effect [19].

By contrast, the United States has made progress but remains constrained by disparities. Despite early adoption and wide availability through initiatives such as the Vaccines for Children program, coverage has plateaued at around 60% for adolescents completing the full series [20]. Uptake is uneven across states and is strongly influenced by socioeconomic status, race, and parental attitudes. Vaccine hesitancy, reinforced by misinformation and inconsistent healthcare provider recommendations, continues to undermine broader impact [21].

In India, groundbreaking clinical trials demonstrated that a single dose of the HPV vaccine provides immune protection not inferior to multi-dose regimens [22]. These results, endorsed by the World Health Organization, have positioned India at the forefront of a paradigm shift that could simplify logistics, reduce costs, and expand access, especially in low- and middle-income settings. Sub-Saharan African countries, supported by Gavi and international partners, have piloted school- and community-based HPV vaccination with encouraging uptake in some regions. However, challenges persist: fragile health infrastructure, rural outreach difficulties, and cultural barriers remain substantial obstacles [23].

Latin America has produced mixed results. Countries such as Mexico and Argentina successfully introduced HPV vaccination in schools and have reported moderate to high coverage in certain cohorts [24]. However, the region as a whole remains heterogeneous, with coverage varying widely according to political stability, health system capacity, and public trust. The COVID-19 pandemic further disrupted delivery, exacerbating vulnerabilities already present in immunization programs [25].

### 3.2. The Brazilian Experience: Achievements and Setbacks

Brazil stands as both a success story and a cautionary tale. In 2014, the country incorporated HPV vaccination into its National Immunization Program (PNI), initially targeting girls aged 9–13 years through school-based campaigns [26]. The results were promising: in the first year of implementation, first-dose coverage exceeded 80%, aligning Brazil with the world’s leading vaccination programs [27]. Public enthusiasm, widespread media coverage, and school participation all contributed to this early momentum.

Yet this trajectory proved fragile. After 2016, coverage rates began to decline, and by 2022–2023, complete vaccination fell below 60% in many states [28]. Several converging factors explain this reversal. First, the dismantling of consistent school-based campaigns reduced opportunities to reach adolescents effectively. Second, vaccine misinformation spread rapidly through social media, with unfounded claims linking the HPV vaccine to infertility, autoimmune diseases, or neurological complications. These rumors, though scientifically baseless, gained traction in communities with limited health literacy and eroded public confidence. Third, the COVID-19 pandemic further disrupted routine immunization: school closures and health system overload diverted attention away from HPV vaccination, creating large cohorts of adolescents who missed their doses [29].

To address these setbacks, Brazil progressively expanded eligibility. Boys were incorporated into the program, followed by immunocompromised individuals, including those living with HIV. Most recently, the Ministry of Health raised the upper age limit for catch-up vaccination, specifically aiming to immunize adolescents and young adults who missed vaccination during the pandemic [27,28]. These adjustments represent an effort to restore coverage and prevent long-term setbacks in cervical cancer prevention.

Historically, Brazil’s cervical cancer screening has been opportunistic, based on cytology every three years for individuals with a cervix aged 25–64 years after two initial annual negatives. In August 2025, the Ministry of Health and INCA launched new national guidelines (Part I) introducing an organized screening program using primary HPV DNA testing within the SUS [30]. Implementation is being phased in and will temporarily coexist with cytology in certain municipalities during the transition period. As HPV-based screening becomes established, public understanding of HPV’s causal link with cervical cancer is expected to improve, which may, in turn, strengthen vaccine acceptance as part of a comprehensive cancer-prevention strategy.

Despite universal access guaranteed by SUS, significant disparities persist. Coverage remains systematically lower in poorer municipalities and in the North and Northeast regions, reflecting structural inequities in healthcare delivery [31]. The Brazilian experience illustrates that strong infrastructure and free provision alone are insufficient: sustaining coverage requires robust, context-sensitive communication strategies, consistent school-based delivery, and active engagement of both healthcare providers and communities.

## 4. Discussion

### 4.1. Vaccine Hesitancy, Misinformation, and Equity

The World Health Organization has identified vaccine hesitancy as one of the ten greatest threats to global health [32], and HPV vaccination exemplifies this challenge. Globally, hesitancy arises from diverse concerns: exaggerated fears of adverse events, cultural or religious opposition to vaccines associated with sexual activity, and distrust of governmental or scientific institutions [33]. In the United States and parts of Europe, parental resistance to vaccinating preadolescents has slowed progress despite ample vaccine availability [34]. In low-resource settings, hesitancy often interacts with logistical barriers, magnifying inequities in access [35].

Brazil’s experience mirrors these global dynamics but also presents unique features. Qualitative research has shown that misinformation, particularly spread through social media platforms, has been a major driver of declining HPV vaccine coverage [36]. Rumors of infertility or severe side effects, although systematically refuted by epidemiological studies, have resonated in communities with lower health literacy. In some municipalities, adolescents and parents actively refused the vaccine, undermining local immunization efforts [37].

Equity further complicates the scenario. While wealthier regions and urban centers often sustain higher coverage, marginalized groups—including populations in rural areas, Indigenous communities, and the urban poor—remain disproportionately under-vaccinated [30]. This inequity perpetuates higher cervical cancer incidence and mortality among individuals with a cervix who are already burdened by social vulnerability [31,38].

**Figure 2:**
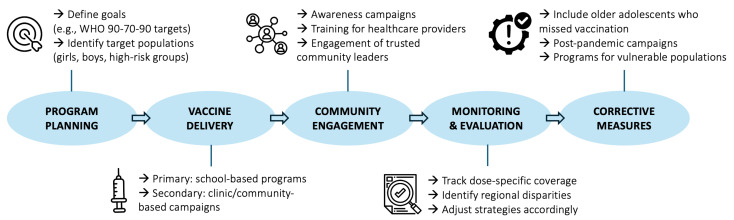
Conceptual framework for successful HPV vaccination programs, developed by the authors based on WHO guidance [13,39].

Globally, successful experiences emphasize that combating hesitancy requires more than scientific evidence. Proactive communication campaigns, school and community engagement, transparent monitoring of safety, and partnerships with trusted leaders have proven essential to sustaining confidence (Figure 2). For Brazil and other middle-income countries, the challenge is twofold: correcting misinformation while simultaneously ensuring equitable access to vaccination.

### 4.2. Policy Implications and Future Directions

Global policy now converges on early adolescent HPV vaccination—ideally before sexual debut—using simplified, equitable, and gender-neutral strategies. The WHO endorses single-dose schedules for girls aged 9–14 years, citing non-inferior protection and clear programmatic advantages, while two doses remain acceptable where feasible [38]. Many countries retain catch-up through mid-adolescence and increasingly vaccinate boys to accelerate herd effects and prevent male HPV-related cancers [40]. In the United States, routine vaccination is advised at 11–12 years with catch-up through 26 years, and selective use up to age 45 based on shared decision-making, reflecting lower cost-effectiveness in older adults [41]. Immunocompromised individuals, including PLHIV, should continue multi-dose schedules due to potentially reduced immunogenicity [42].

Building on this global guidance, several high- and middle-income countries have already transitioned to single-dose implementation in alignment with the WHO Strategic Advisory Group of Experts (SAGE) 2022 recommendation [38]. These coordinated policy shifts align with the WHO SAGE 2022 position endorsing single-dose schedules for adolescents, now operational in Australia (from February 2023) and the United Kingdom (from September 2023) [38,41]. Australia introduced a one-dose regimen of Gardasil 9 for adolescents in February 2023 [40], while the United Kingdom adopted a single-dose schedule for its school-based and GBMSM programs in September 2023 [41], following its earlier transition to Gardasil 9 in July 2022 [42]. Comparable policy shifts have occurred in Ireland (national school-based program, 2023) [43], are being implemented across selected Canadian provinces and territories (from 2024) [44], and are expanding to several autonomous regions of Spain during 2024–2025 [45]. In Latin America, Brazil—one of the first middle-income countries to adopt nationwide HPV vaccination—has maintained gender-neutral coverage under its National Immunization Program and is currently evaluating the adoption of the single-dose regimen within its updated Technical Note No. 41/2024 [28]. Collectively, these coordinated actions reflect a growing international consensus that single-dose HPV vaccination offers a durable, cost-effective, and programmatically feasible strategy to sustain high coverage and accelerate progress toward cervical cancer elimination.

Coverage remains heterogeneous. School-based programs in Australia and the United Kingdom achieve high completion and have produced measurable declines in HPV infection, genital warts, high-grade lesions, and cervical cancer [17,18,19,20]. By contrast, the United States shows uneven uptake with persistent disparities [20,21,46], while many LMICs face logistical and supply barriers that limit performance despite political commitment [23,25,38]. WHO’s single-dose guidance is expected to enhance feasibility and cost-effectiveness in such settings, especially amid post-COVID-19 recovery efforts [38,47,48].

Brazil’s National Immunization Program (PNI), launched in 2014, initially achieved >80% first-dose coverage but later declined below 60% due to misinformation, weakened school delivery, and pandemic disruptions [28]. Recent updates expand eligibility to boys, immunocompromised groups, and older adolescents, aligning with global policy [28,49,50].

Three prophylactic VLP vaccines underpin global programs—bivalent (HPV16/18), quadrivalent (adding 6/11), and 9-valent (adding 31/33/45/52/58)—all highly immunogenic and protective against high-grade lesions [51,52]. Beyond the pivotal efficacy trials [51], long-term follow-up confirms the durability of 9-valent HPV vaccine protection. In adolescents vaccinated at 9–15 years, 8-year data show sustained immunogenicity and protection against persistent infection [53]. Among Scandinavian individuals with a cervix, 12-year results demonstrate continued effectiveness against targeted HPV types [54]. Newer licensed products (Cecolin, Walrinvax/HPV-2, Cervavac, and Cecolin-9) are broadening access in China and India, with WHO prequalification expanding procurement options [38].

Research on next-generation vaccines seeks broader coverage (L2-based platforms) [55], therapeutic formulations targeting E6/E7 for CIN2/3 [56], and innovative delivery systems such as thermostable or microneedle formulations to simplify logistics [57,58]. Although maximal benefit accrues with pre-exposure vaccination, older adolescents, MSM, transgender, and immunocompromised populations continue to benefit [59,60].

Post-treatment vaccination after HSIL/CIN^2+^ is emerging as a cost-effective adjuvant strategy: meta-analyses show 57–74% recurrence reduction, improved HPV clearance, and favorable economic profiles in multiple countries [39,61,62,63,64,65,66]. Household or school catch-up campaigns remain vital to restore herd protection [67].

As vaccinated cohorts reach screening age, cervical cancer prevention will increasingly depend on coordinated optimization between vaccination and HPV-based screening. Modelling studies suggest that high vaccination coverage permits safely increasing the starting age of screening and reducing lifetime screening frequency without compromising cancer prevention [68]. The introduction of self-sampling for HPV testing offers a complementary strategy to expand coverage, particularly among under-screened individuals with a cervix and underserved populations in rural or low-resource areas. Integrating vaccination and screening within primary care and digital registries will be essential to track outcomes, monitor effectiveness, and guide evidence-based adjustments in national prevention programs. Such coordinated planning ensures cost-effectiveness, minimizes unnecessary interventions, and maximizes population-level impact toward achieving the WHO’s cervical cancer elimination goals.

Looking ahead, the consolidation of single-dose schedules, gender-neutral programs, and integration with HPV-based screening will define the next phase of cervical cancer prevention. Addressing vaccine hesitancy—one of WHO’s top global health threats [32]—requires proactive communication, engagement with trusted messengers, and transparency on safety [69]. Strengthened supply chains, flexible catch-up policies, and preparedness for future crises will be essential to sustain progress.

Programmatic strategies to improve HPV vaccination coverage include co-administration with other adolescent vaccines such as Tdap and MenACWY, which have demonstrated non-inferior immunogenicity and acceptable safety profiles [70,71]. Offering multiple vaccines during the same visit reduces missed opportunities and improves series completion [72]. Additionally, initiating HPV vaccination at age 9—rather than 11–12—has been associated with higher on-time completion and greater parental acceptance by decoupling the vaccine from discussions of sexual activity [73]. Together, these approaches represent practical levers to normalize HPV vaccination, enhance uptake, and strengthen adolescent immunisation programmes.

In practical terms, restoring school-based HPV vaccination and implementing community catch-up campaigns for missed cohorts remain priorities to rebuild herd protection. Integrating HPV vaccination with reproductive health and primary care services can enhance programmatic efficiency, while gender-neutral strategies promote equity and maximize population impact. Expanding digital surveillance against misinformation and fostering intersectoral partnerships between education and health sectors will further support vaccine confidence and sustainability.

The experiences of the eight countries analyzed reveal practical lessons that extend beyond HPV vaccination itself. The consistent success of school-based, publicly funded programs in Australia, the United Kingdom, Canada, and Spain highlights the importance of accessible delivery platforms and gender-neutral communication. In contrast, challenges observed in Brazil, India, and the United States underscore the need for sustained investment in vaccine confidence, digital misinformation control, and post-pandemic recovery of coverage. China’s rapid expansion of domestic production and pilot single-dose implementation further illustrates how policy innovation can promote equity and affordability. Together, these findings suggest that adapting proven strategies to national contexts—particularly those emphasizing equity, communication, and delivery—can accelerate the global goal of cervical cancer elimination.

Ultimately, sustainable success will depend on aligning scientific innovation with resilient delivery systems and public trust. For Brazil and other middle-income nations, this alignment will determine whether the WHO 90-70-90 elimination targets can be achieved within the coming decades.

## 5. Conclusions

HPV vaccination represents one of the most powerful tools ever developed for cancer prevention. In less than two decades, real-world data from countries such as Australia and the United Kingdom have confirmed its ability not only to reduce HPV infections and precancerous lesions but also to lower the incidence of invasive cervical cancer. At the same time, experiences from the United States, India, Latin America, and sub-Saharan Africa underscore both the opportunities and the challenges inherent in achieving widespread, equitable implementation.

The Brazilian case illustrates the paradox faced by many middle-income countries. Despite a strong public health infrastructure and the initial success of school-based vaccination campaigns, coverage has declined in recent years due to misinformation, logistical barriers, and the impact of the COVID-19 pandemic. The subsequent expansion of eligibility to boys, immunocompromised populations, and older adolescents and the recent adoption of national guidelines for organized HPV-based screening within SUS are important corrective steps, but success will depend on sustained communication strategies, systematic catch-up efforts, and renewed political commitment.

Looking forward, the adoption of simplified schedules such as single-dose vaccination, the integration of gender-neutral programs, and earlier initiation from age 9, alongside routine co-administration with other adolescent vaccines, and the development of next-generation vaccine platforms are poised to strengthen global cervical cancer prevention. Yet these scientific advances will only achieve their full potential if they are coupled with equitable access, integrated HPV-based screening programs, robust delivery systems, and effective measures to combat vaccine hesitancy.

Ultimately, the path to eliminating cervical cancer as a public health problem requires more than biomedical innovation. It demands coordinated policies, societal trust, and a commitment to reaching the most vulnerable populations. If these conditions are met, HPV vaccination can fulfill its promise as a cornerstone of the WHO’s 90-70-90 elimination strategy, transforming the future of cervical cancer prevention and reproductive health in Brazil and worldwide.

## Figures and Tables

**Table 1:** Global Overview of National HPV Immunization Programs: Vaccine Platforms, Coverage, Equity, and Public Health Impact.

Country	Program Start	Target Age	Delivery Strategy	Vaccine Type	1st Dose Coverage (%)	Full Series Coverage (%)	Reported Impact	Equity/Funding	Source
United States	2006	11–12 years	Clinic-based	Quadrivalent/9-valent (Gardasil^®^, Gardasil 9^®^)	~60%	~60%	Moderate progress; persistent geographic and sociodemographic gaps	Publicly funded through Vaccines for Children (VFC); gender-neutral (boys and girls)	CDC, National Immunization Survey–Teen, 2023
Australia	2007	12–13 years	School-based	Quadrivalent/9-valent (Gardasil 9^®^, single-dose since 2023)	>80%	>70%	Dramatic decline in HPV 16/18 infection and high-grade lesions; early evidence of cervical cancer reduction	National Immunisation Program; gender-neutral; single-dose policy since February 2023	Australian Department of Health, 2023
United Kingdom	2008	12–13 years	School-based	Gardasil 9^®^ (nonavalent, single-dose since 2023)	>85%	>80%	86% reduction in HPV 16/18 among <21 years; projected elimination of cervical cancer within decades	NHS-funded; gender-neutral since 2019; single-dose Gardasil 9 since September 2023	UK Health Security Agency, 2023
Canada	2007	9–26 years (routine 9–15)	School-based (primary), community clinics	Quadrivalent/9-valent (Gardasil^®^, Gardasil 9^®^)	80–90% (first dose, girls)	70–85%	Sharp decline in HPV prevalence and anogenital warts; early evidence of reduced cervical intraepithelial neoplasia (CIN^2+^)	Publicly funded via provincial programs; gender-neutral since 2015; school-based coverage > 85% in most provinces	Public Health Agency of Canada, Immunization Coverage Reports, 2023
Spain	2007	12 years (girls), 12–18 years (catch-up; boys since 2023)	School-based	Bivalent/9-valent (Cervarix^®^, Gardasil 9^®^)	~90% (girls)	~80%	Significant reduction in HPV 16/18 infection; rapid uptake of gender-neutral vaccination since 2023	Fully funded under the National Health System; regional management by autonomous communities	Spanish Ministry of Health, Vaccination Calendar, 2024
China	2016 (national approval)	9–14 years (recommended)	School/clinic	Cecolin^®^ (bivalent, WHO PQ 2021); Walrinvax^®^/HPV-2 (bivalent, WHO PQ 2024); Cecolin-9^®^ (nonavalent, 2025)	Expanding (20–60%)	Not available	Rapid uptake in pilot provinces; increasing domestic vaccine availability and single-dose implementation	Public–private partnership; domestic supply ensures affordability	National Health Commission of China, 2025; WHO PQ listings, 2024
India	2008 (state-level pilots); national rollout 2024	9–14 years	School/clinic	Cervavac^®^ (quadrivalent); Cecolin^®^ (bivalent)	Variable (pilot states 60–80%)	Variable	Single-dose evidence supports durable protection; national scale-up underway	Public programs expanding through state immunization systems; primarily girls 9–14 years	WHO/UNICEF Joint Reporting Form, 2023; Serum Institute of India, 2024
Brazil	2014	9–13 years (girls, later boys)	School-based	Quadrivalent (Gardasil^®^) via PNI	>80% (first dose)	<60%	Early success followed by decline due to misinformation and pandemic disruptions; recovery underway with expanded eligibility	National Immunization Program (SUS); gender-neutral since 2017; publicly funded	Brazilian Ministry of Health, PNI Report, 2024

Data derived from WHO/UNICEF coverage estimates, PAHO regional reports, and national immunization plans. WHO-prequalified vaccines include Cervarix^®^ (GSK (London, UK)), Gardasil^®^/Gardasil 9^®^ (Merck/MSD (Rahway, NJ, USA)), Cecolin^®^ (Innovax, Xiamen, China), and Walrinvax/HPV-2^®^ (Walvax, Kunming, China). Cervavac^®^ (Serum Institute of India (Pune, India)) and Cecolin-9^®^ (Innovax, Xiamen, China) are nationally licensed with WHO PQ in process.

## Data Availability

Not applicable.
